# Epilepsy, Immunity and Neuropsychiatric Disorders

**DOI:** 10.2174/1570159X20666220706094651

**Published:** 2023-06-15

**Authors:** Francesco Fortunato, Alessia Giugno, Ilaria Sammarra, Angelo Labate, Antonio Gambardella

**Affiliations:** 1 Department of Medical and Surgical Sciences, Institute of Neurology, Magna Graecia University, Catanzaro, Italy;; 2 BIOMORF Department, Neurology Unit, University of Messina, Messina, Italy;; 3 Institute of Molecular Bioimaging and Physiology, National Research Council, I-88100 Catanzaro, Italy

**Keywords:** Epilepsy/seizures, immunity, autoimmune encephalitis_,_ neuro-inflammation, neuropsychiatric disorders, immunotherapy

## Abstract

Several studies have focused on the emerging role of immunity and inflammation in a wide range of neurological disorders. Autoimmune diseases involving central nervous system share well defined clinical features including epileptic seizures and additional neuropsychiatric symptoms, like cognitive and psychiatric disturbances. The growing evidence about the role of immunity in the pathophysiologic mechanisms underlying these conditions lead to the concept of autoimmune epilepsy. This relatively-new term has been introduced to highlight the etiological and prognostic implications of immunity in epileptogenesis. In this review, we aim to discuss the role of autoimmunity in epileptogenesis and its clinical, neurophysiological, neuroimaging and therapeutic implications. Moreover, we wish to address the close relationship between immunity and additional symptoms, particularly cognitive and psychiatric features, which deeply impact clinical outcomes in these patients. To assess these aspects, we first analyzed Rasmussen’s encephalitis. Subsequently, we have covered autoimmune encephalitis, particularly those associated with autoantibodies against surface neuronal antigens, as these autoantibodies express a direct immune-mediated mechanism, different from those against intracellular antigens. Then, we discussed the connection between systemic immune disorders and neurological manifestations. This review aims to highlight the need to expand knowledge about the role of inflammation and autoimmunity in the pathophysiology of neurological disorders and the importance to early recognize these clinical entities. Indeed, early identification may result in faster recovery and a better prognosis.

## 1 INTRODUCTION

Growing evidence highlights the crucial role of autoimmunity and inflammation in the pathogenesis and management of epilepsy with neuropsychiatric comorbidity [1]. According to the latest classification by the International League against epilepsy (ILAE), an “*autoimmune etiological category*” has been introduced [2], recognizing the connection between autoimmunity, inflammation and epilepsy [1-3]. Several factors supported this link. First of all, autoimmunity seems to be the main etiology in about 15-20% of epilepsies with unknown etiology [3].

The wide spectrum of symptoms in autoimmune encephalitis (AIE) and autoimmune diseases involving the central nervous system (CNS) provides new insight into the striking connection between autoimmunity, epileptogenesis, and neuropsychiatric disorders [1-3]. Autoimmune epilepsy is suspected in patients with seizures and clues for autoimmunity like the presence of neuronal autoantibodies (Auto-Abs) or inflammatory changes in serum, cerebrospinal fluid (CSF), or brain magnetic resonance imaging (MRI) [4, 5]. Interestingly, neural Auto-Abs are detected in about 36% of patients with epilepsy refractory to anti-seizure medications (ASMs) and 75% of them showed a significant reduction in seizure frequency after immunotherapy [6]. Several studies *in vivo* and *in vitro* further support the pathophysiological link between inflammation, autoimmunity and epileptogenesis [7, 8]. Firstly recognized in 1958, Rasmussen encephalitis (RE) represents a chronic neurological disorder characterized by unilateral inflammatory process of the cerebral cortex [9]. In a broad spectrum, autoimmune epilepsy includes a growing number of epileptic disorders with autoimmune etiology as also autoinflammatory diseases presenting with seizures or status epilepticus (SE). An early diagnosis of autoimmune epilepsy deeply impacts therapy and seizure outcomes since patients can benefit from immunomodulant treatment [10]. When clinical and serological clues suggest an autoimmune basis for epilepsy refractory to ASMs, early immunotherapy can improve seizure outcome [10]. Recently, a scoring system called the “*antibody prevalence in epilepsy of unknown etiology score*” (APE) has been proposed to quantify the risk for autoimmune epilepsy and to guide the diagnosis [11]. This review discusses the role of autoimmunity and of inflammation in RE, AIE and systemic autoimmune diseases with CNS involvement, manifesting with seizures and neuropsychiatric symptoms.

## 2 RASMUSSEN’S ENCEPHALITIS

Rasmussen’s encephalitis (RE) is an epileptic syndrome in which there is no proof of a clear autoimmune etiology [9, 12]. It is a rare chronic neurological disorder characterized by asymmetric inflammation of the cerebral cortex with unilateral hemispheric atrophy, drug-resistant epilepsy, progressive hemiparesis, and cognitive impairment [12]. RE is typically a severe condition that affects children, although milder forms and late-onset cases have been reported [13]. Epidemiological data report an incidence of 2.4 per 10^7^ people under 18 years per year [14]. The clinical progression of RE has been classically divided into three phases: a prodromal stage in childhood with low seizure frequency and mild hemiplegia; an acute stage with a high frequency of epileptic seizures, progressive hemiparesis, and cognitive deterioration; a residual stage with permanent neurological deficits and continuing seizures [12]. Epileptic seizures may include varying semiologies in the same patient, including epilepsia partialis continua in about 50% of affected patients [15]. The neuropsychiatric features typically include severe cognitive decline and dysphasia if the dominant hemisphere is affected. A relative nonprogressive form of the disorder which starts in adult life with a milder course and temporal lobe epilepsy (TLE) has been also described [16]. In 2005, the European consensus panel proposed formal diagnostic criteria for RE which include focal seizures with or without epilepsia partialis continua, unihemispheric slowing at electroencephalogram (EEG) and unihemispheric focal cortical atrophy at brain MRI [17]. Long-term MRI features of childhood RE show a more severe progression of unilateral cortical atrophy and grey matter abnormalities compared to the late-onset form [18]. RE’s inflammatory nature is proved by pathological surgical specimens’ which showed cortical inflammation, neuronal loss and microglial nodules with perivascular mononuclear cell infiltration [19]. Although antibodies anti glutamate receptor GluR3 has been considered to have a causative role in RE, as suggested by animal models of disease [20], more recently, the role of these antibodies has been questioned [21]. First, antibodies anti-GluR3 have been detected either in a very small portion of RE patients and other chronic patients with epilepsy [21, 22]. Second, analysis of inflammatory infiltrates in surgical specimens shows a mixed infiltrate of dendritic cells and cytotoxic CD3+/CD8+ T lymphocytes, so that RE is mainly considered a T-mediated disorder [23]. Current studies suggest an adaptive immune mechanism mediated by CD8+ T cells and an innate immune mechanism mediated by activated microglia and neuroglia [24]. However, a specific self-antigen in RE patients has never been confirmed [25]. Thus, RE does not fulfill Koch/Witebsky’s postulates [26]. Similar to clinical evolution, RE also evidences a neuropathological progression, dealing with four stages with the progressive cortical injury sustained by T-lymphocyte and neuroglial activation [19]. A retrospective series of 160 RE cases showed that perinatal complications and other autoimmune conditions (like scleroderma, uveitis, and chorioretinitis) might predispose to RE [27]. Treatment in RE syndrome aims to reduce seizure frequency and improve motor and long-term cognitive outcomes. Seizures in RE tend to be very refractory to anti-seizures medications (ASMs). Immune treatment, as steroids, plasmapheresis and anti-CD20 monoclonal antibody as rituximab proved to only temporary beneficial effects on seizure frequency [28, 29]. In late-onset RE, long-term chronic immunotherapy with corticosteroids, intravenous immunoglobulins (IVIGs) and T-cell inactivating drugs, as tacrolimus and azathioprine (AZA), exerted a mild effect [30]. A recent study in a cohort of 30 pediatric RE patients demonstrated the effectiveness of AZA in slowing disease progression, particularly in the early stages [31]. Despite this slight evidence, the surgical hemispherectomy or hemispherotomy often remains the only definitive therapeutic option in refractory cases [32]. A follow-up study of 40 RE patients who received hemispherotomy reported an improvement in cognitive dysfunctions after a hemispherectomy: seizure duration and age at surgery were associated with post-operative cognitive outcomes [33]. Moreover, the identification of T-cell involvement in the RE pathogenesis, with a specific cytokine pattern, opens a window for T-targeted therapies and biologic drugs in the treatment [34].

## 3 AUTOIMMUNE ENCEPHALITIS

AIE represents a relatively recent clinical entity characterized by the association of cognitive symptoms, psychiatric disturbances, and epileptic seizures, according to diagnostic criteria [35]. Although traditionally infectious encephalitis are believed to be more frequent, AIE triplicated in the last years, also due to more accurate diagnostic methods [3, 36]. AIE and infectious encephalitis overlap in incidence and prevalence, interesting about 13.7/100000 and 0.8/100000 persons per year, respectively. Thus, AIE constitutes a health and economic burden [3, 36]. Interestingly, herpes simplex encephalitis may constitute an immunological trigger for AIE, occurring within three months of treatment with acyclovir in 27% of cases. This association could reflect an immune-mediated link between infectious and autoimmune encephalitis [37]. AIE seems to affect predominantly African-American ethnicity [3] and female gender [38, 39], although conflicting data exist in the literature [40, 41]. However, the lack of specificity in AIE clinical features probably underestimates the real incidence and prevalence [3, 35]. Traditionally, patients with an age <45 years seem to be more likely to develop AIE [38]. Nevertheless, accordingly to a recent study, AIE displays a bimodal incidence, showing the first peak at 11-20 years and the second one at 61-70 years [40]. Based on the type of neuronal antibody and correspondent immune-mediated mechanism, AIE may be related to surface neuronal auto-Abs and auto-Abs directed against intracellular antigens [35]. The former recognizes a B-mediated immune system mechanism and underlying autoimmune etiology in most cases [35]. The latter presents a T-cells mediated immune process and could be related to a neoplasm’s presence [35]. Their clinical hallmarks include subacute cognitive impairment (CI), epileptic seizures, psychiatric manifestations, movement disorders, and dysautonomia [3]. In AIE, epilepsy is refractory to ASMs in about 79.5% of cases [35]. The prognosis is favorable in about 80% of subjects [39, 42], and patients may benefit from early immunotherapy [35]. However, in about 23-30% of cases, AIE might be associated with tumors [41-43]. In such a case, treatment of the underlying neoplasm is essential to obtain remission of AIE. Some factors may correlate with a bad prognosis, such as male gender, advanced age at onset, presence of antibodies on CSF, psychiatric symptoms, movement disorders, impaired consciousness, central hypoventilation, status epilepticus (SE), admission in intensive care unit (ICU), tumors, and higher neutrophil-to-lymphocyte ratio (NLR) [39, 40, 43, 44]. Moreover, Zeng *et al.* demonstrated that NLR is a biomarker of disease progression in AIE [45]. The rate of mortality ranges from 2.65% to 14.5% [36, 38, 39, 41]. The most effective treatment in AIE is immunotherapy [46-48] with corticosteroids, IVIGs or plasmapheresis, second-line and third-line treatment, including monoclonal antibodies [48].

### 3.1 Autoimmune Encephalitis and Epilepsy

The emerging term “autoimmune epilepsy” highlights the role of the immune system and its dysregulation in epileptogenesis, leading to seizures in AIE. Both animal and autoptic studies enhance the immune system’s involvement in AIE, detecting inflammatory changes in the brain of patients affected by anti-N-methyl-D-aspartate receptor (NMDAR-IgG) encephalitis as well as chronic lymphocytic infiltrates with gliosis and neuronal loss in those cases related to onconeural antibodies [49-51]. In AIE, epileptic seizures may manifest as both focal and generalized and represent a core clinical symptom. Particularly, the prototype of AIE corresponds to limbic encephalitis (LE), presenting with rapidly progressive or subacute (less than three months) deficit of working memory, epileptic seizures, and psychiatric symptoms, closely reflecting the limbic system involvement [35]. AIE could present as SE. Interestingly, about 2.5% of cases of SE seem to have an immune-mediated etiology [52]. Overall, the prevalence of autoimmune epilepsy is unclear [39, 53]. Around 5% of patients with TLE depict an autoimmune etiology, whereas autoimmune etiology is less represented in patients with TLE and hippocampal sclerosis (HS) [39]. AIE may also present acute symptomatic seizures with highly heterogeneous semiology, often resolved after prompt immunotherapy or ASMs [1]. However, the risk of developing chronic, long-standing epilepsy varies widely according to the targeted autoantigen and the timing of immunotherapy. Patients with AIE associated with auto-Abs against intracellular antigens are more likely to develop chronic epilepsy, compared to those with AIE associated with surface ones. Furthermore, other factors, such as presence of hippocampal atrophy on MRI, interictal epileptiform discharges at EEG and immunotherapy delay, increase the risk of developing chronic epilepsy in AIE [50]. In such a case, the autoimmune and structural etiology coexist [39]. Long-term prospective studies are warranted to better comprehend risk factors for chronic epilepsy in AIE and thus influence clinician decision strategies. Epileptic seizures might have different semiology, including facio-brachial dystonic seizures (FBDS) and pilomotor seizures, such as in anti- leucine-rich glioma inactivated-1 (LGI-1-IgG) AIE [49]. EEG lacks a specific pattern, although “extreme-delta brush” (EDB) seems to identify anti-NMDAR-IgG AIE [54, 55]. In these forms, epileptic seizures are often refractory to ASMs and may respond to immunotherapy. In AIE, epileptic seizures are strictly connected to inflammatory changes and neuronal loss. Interestingly, inflammation and neurodegeneration appear to be implied in the development of HS, correlating to elevated CSF t-tau levels in patients with AIE [56].

### 3.2 Autoimmune Encephalitis and Cognitive Impairment

CI is a core clinical feature of AIE, often presenting as a subacute loss of short-term and/or working memory, associated with psychiatric changes and new-onset neurological signs [35]. Short-term and episodic memory is the most involved cognitive domains in LE and AIE, especially those associated with anti-NMDAR-IgG and Voltage-gated potassium channel (VGKC-IgG) antibodies, reflecting the expression of the correspondent antigens on the hippocampal structures [57]. However, extratemporal cognitive functions may also be involved, particularly in patients with rapidly progressive CI, possibly mimicking Creutzfeldt-Jacob disease. In these cases, inflammation may lead to functional damage in the early stage of disease, subsequently evolving into structural damage and a chronic CI with disease progression [57]. Thus, neuropsychological evaluation is essential in differential diagnosis and early identification of CI in clinical practice. Indeed, as recently observed, CI, affecting about 50% of patients with autoimmune epilepsy and preferentially involving executive and memory domains, correlates to autoantibodies and the acute phase of AIE [58, 59]. Thus, the concept of CI as merely short-term memory loss may be reductive considering the wide spectrum of cognitive domains involved. CI is often subtle and subacute, sometimes representing the first or unique sign of AIE. In about 40% of cases, the rapidly progressive CI has an autoimmune etiology [57]. Furthermore, CI may also be chronic and hard to differentiate from neurodegenerative cognitive decline, leading to misdiagnosis and mistreatment [59]. A recent study demonstrates that 16-38% of patients with AIE, especially when associated with anti-NMDAR-IgG, anti-LGI-1-IgG, anti- gamma aminobutyric acid-B receptor-IgG (GABABR-IgG) and anti-Contactin-Associated Protein-Like 2 (Caspr2) auto-Abs, might mimic dementia, also manifesting additional clinical features, such as epileptic seizures and psychiatric symptoms. In these cases, CSF analysis and brain MRI may be helpful to rule out neurodegenerative disorders, such as prion diseases. However, these exams may not show abnormalities, delaying the correct diagnosis and treatment [59, 60]. Anti-NMDAR-IgG encephalitis represents a model of CI in AIE. Interestingly, patients with this condition exhibit altered connectivity of the left insula with the inferior parietal lobe and enhanced connectivity with the superior parietal lobe. Insula is considered an intermediate pathway for several cognitive processes, such as attention and working memory [61]. Immunotherapy plays a key role in achieving remission of cognitive symptoms and avoiding long-term sequelae, which may involve about 20% of patients [62].

### 3.3 Autoimmune Encephalitis and Psychiatric Symptoms

The dysregulation of the immune system in psychiatric disorders has been postulated based on the increasing observation of psychiatric disease in immune-mediated neurological disorders, as AIE. Indeed, about 83% of AIE patients may develop psychiatric symptoms, such as delusions, hallucinations, and personality and behavior changes [35, 63, 64]. According to a recent Consensus, the association of additional features, such as prodromal infectious illness, new-onset headache, rapid progression of the disease, scarce or adverse response to antipsychotic drugs and neurological manifestations, including seizures, CI, movement disorders, dysautonomia, suggested to consider an autoimmune etiology of psychiatric disorders [65]. Based on the emerging role of inflammation in the pathophysiology of psychiatric disorders, a recent study characterized the immune cell profile of primary psychotic disorders compared with secondary psychosis [66]. According to their findings, patients with psychosis and anti-NMDAR-IgG AIE share blood-brain barrier (BBB) disruption with raised CSF proteins [66]. However, they show different immunological profiles, given that patients with anti-NMDAR-IgG AIE demonstrate higher levels of monocytes in CSF, compared to those with primary psychosis [66]. Typically, immune-mediated psychiatric symptoms show a better response to immunotherapy and worse to anti-psychotic drugs [63].

### 3.4 NMDAR-IgG Autoimmune Encephalitis

First described in 2005 [67], anti-NMDAR-IgG AIE is a prototype of AIE and fulfills Koch/Witebsky’s postulates [26, 49, 68, 69]. Anti-NMDAR-IgG AIE largely affects the female gender [48, 67, 68, 70] and young-adult patients, with a mean age at an onset of 21 years [48, 70]. Clinical presentation includes the subacute onset of psychiatric symptoms, memory and speech disturbances, and neurological features, such as epileptic seizures, movement disorders, autonomic dysfunction, decreased level of consciousness, and central hypoventilation [49, 70, 71]. Patients may also experience prodromal symptoms, such as viral-like disease or headache. This heterogeneous clinical picture reflects the different distributions of NMDAR in the CNS. The underlying mechanism is mediated by IgG antibodies against the N-terminal of the NR1 subunit of NMDAR, which induces capping, cross-linking and internalization of NMDA receptors [72, 73]. Although IgA and IgM NMDAR antibodies have also been identified, they are not pathogenic [73]. The hallmark of psychiatric presentation is the polymorphic coexistence of several features, such as anxiety, agitation, disinhibition, abnormal behavior, hallucinations, and delusions, mimicking the first episode of psychosis [74, 75]. Thus, patients may initially present to psychiatric services in 60-70% of cases [76]. The predominant psychiatric and memory dysfunctions in anti-NMDAR-IgG AIE, due to the reduction of synaptic NMDAR, confirm a common pathophysiologic mechanism between anti-NMDAR-IgG AIE and psychiatric diseases, as postulated by the theory of NMDA-hypofunction in schizophrenia [74]. Psychiatric and memory deficits may also reflect a partial reduction of the GluN1 subunit of NMDAR in GABAergic interneurons of the cortico-limbic pathway [74]. Moreover, the neuropsychiatric symptoms correlate to the disruption of BBB with consequent higher CSF protein and IgG intrathecal synthesis, especially in the acute phase of the disease [69]. The BBB disruption might be caused by a greater immune response, correlating with a higher admission rate in the ICU, a less favorable outcome, and elevated NLR [69, 70]. Patients with anti-NMDAR-IgG AIE may also experience movement disorders, such as involuntary oral movements, orofacial dyskinesia, choreoathetosis [49]. The diagnosis of anti-NMDAR-IgG AIE is supported by neurophysiological assessment and neuroimaging. In about 97% of patients, EEG detects abnormal findings, such as epileptic discharges, slow activity, beta fast activity, background slowing, and periodic discharges [77]. EDB has been proposed as a pathognomonic EEG feature [54, 55, 78]. Brain MRI is abnormal in 30-50% of cases [49, 71], displaying a “diffuse encephalopathy”, cerebral and cerebellar atrophy, medial temporal lobe abnormalities or altered signals in other brain areas [70, 71]. ^18^F-fluorodeoxyglucose-positron emission tomography (^18^F-FDG-PET) may also support the diagnosis and monitoring of disease. A distinctive ^18^F-FDG-PET pattern in NMDAR-IgG AIE is the “*anterior-posterior decreasing FDG uptake gradient*”, with relative anterior hypermetabolism and posterior hypometabolism [79]. The prognosis is largely influenced by ICU admission, early immunotherapy and tumour removal [49], with a good prognosis in about 90% of patients [49, 70]. Epileptic seizures may benefit from immunotherapy, whereas ASMs are considered an add-on treatment [78]. CI and neuropsychiatric dysfunction also display a favorable outcome after immunotherapy, although 8% of patients manifest long-term cognitive dysfunction [80] and 17.% might experience clinical relapses [71]. Furthermore, anti-NMDAR-IgG AIE is associated with a mortality rate of 7-10% [69, 71]. According to a recent study, the presence of fever, generalized seizures, altered consciousness, and elevated NLR correlates with a worse outcome [70]. Interestingly, in 3% of cases, NMDAR-IgG antibodies may coexist with other auto-Abs, including glial, as anti-myelin oligodendrocyte glycoprotein, anti-Acquaporin4, anti-Glial Fibrillary Acidic Protein, and neuronal antibodies, anti-GABAAR-IgG and anti-GABABR-IgG. The concomitant anti-glial positivity might explain the demyelinating features in a subset of patients with anti-NMDAR-IgG encephalitis [81, 82]. The co-occurrence of more than one anti-Abs seems to be related to older patients, shorter duration of symptoms at onset, less typical symptoms, and CSF pleocytosis, leading to a less favorable outcome [81]. Moreover, these patients demonstrate a higher rate of MRI abnormalities, admission in ICU, and concomitant tumor [81, 82]. Anti-NMDAR-IgG AIE may associate with teratomas, especially in female patients aged 12 years or older [49]. In such cases, surgery may contribute to resolving clinical manifestations. Even if a first evaluation does not detect a neoplasm, an extensive screening may be taken into account based on the patient’s age and gender [49].

### 3.5 Anti-LGI-1-IgG Autoimmune Encephalitis

LGI-1-IgG encephalitis is an immune-mediated disorder often presenting as the subacute onset of memory, epileptic seizures, cognitive and behavior disturbances, with a good response to immunotherapy. LGI-1-IgG AIE largely affects male patients with a mean age of 61-64 years [46, 83]. The higher prevalence of HLA-DR7 and HLA-DRB4 in patients with anti-LGI-1-IgG AIE supported the immune-mediated etiology [46]. Its main clinical features reflect the ubiquitous expression of LGI-1 proteins, particularly in the hippocampus and temporal cortex. Thus, the clinical presentation may include limbic encephalitis, Morvan syndrome and epileptic seizures [46, 83]. Patients often manifest cognitive dysfunction (42%). Epileptic seizures often involve about 90% of patients. Focal seizures can include impaired awareness, autonomic or gelastic aura and tonic-clonic seizures [46]. FBDS are the pathognomonic type of seizures, although it is still questioned whether this form constitutes an epileptic seizure or dystonia [46]. FBDS consists of recurrent and ipsilateral spasms of the upper limb, face and leg, lasting less than three seconds. About 47% of patients depict FBDS at a higher daily frequency [46]. About 90-97% of patients develop memory and behavior abnormalities, with apathy and disinhibition (53%). However, EEG could document paroxysmal changes in frontal, temporal and parietal regions [46]. Other clinical manifestations may include sleep disturbances, autonomic dysfunction, mainly hyperhidrosis, movement disorders, such as chorea, and bulbar symptoms of myasthenia gravis at the onset. About 65% of patients exhibited hyponatremia [46, 83, 84]. This latter clinical feature reflects the altered secretion of antidiuretic hormone, probably due to the binding of LGI-1-IgG antibody to hypothalamic paraventricular nucleus neurons [46]. In anti-LGI-1-IgG AIE, cognitive dysfunction, often rapidly progressive, more often involves memory domains, especially short-term memory [83], and executive functions, semantic and phonemic fluency [78]. Although the widely known response to immunotherapy, some patients might experience moderate or severe cognitive impairment after two years from the clinical onset [83]. About 75% of patients depicted unremarkable CSF cell count and proteins [46]. According to recent evidence, LGI-1-IgG antibodies are more often detected in serum and less frequently in CSF. Whether serum and CSF titers do not influence prognosis generally, the LGI-1-IgG antibodies positivity on CSF appears to predict a worse outcome [85]. Brain MRI could demonstrate a hippocampus swelling and T2 hyperintense signal with a normal or reduced volume [46]. Subsequently, patients may develop hippocampal atrophy or sclerosis, with a characteristic spared CA1 subfield and involvement of CA3 subfield, implicated in memory encoding [86]. Patients with FBDS might exhibit T1 and T2 hyperintensities in the basal ganglia with a reduced pallidum volume, suggesting a close relationship between alterations of these structures and FBDS [78]. ^18^F-FDG-PET is more sensitive than MRI, particularly in the early diagnosis of the disease [87, 88]. The motor cortex could show a functional damage with a hypermetabolism, without any structural impairment [87]. ^18^F-FDG-PET further strengthens the involvement of these structures, demonstrating a hypermetabolic pattern in basal ganglia (82%), often related to FBDS, and in medial temporal lobes (68%) [87]. Thus, the main targets of anti-LGI-1-IgG antibodies are the motor and temporal cortex, especially the hippocampus [88, 89]. Interestingly, ^18^F-FDG-PET may also help to monitor response to immunotherapy and disease progression [87]. Immunotherapy seems to be more useful to reach seizure freedom in epileptic seizures than ASMs. Although the most utilized ASM is levetiracetam, it may enhance behavioral and psychiatric symptoms in patients. Carbamazepine, lacosamide and oxcarbazepine are the greatest choices in focal seizures [78]. Patients need a combination of ASMs and immunotherapy associated with high-dose corticosteroids and/or intravenously immunoglobulins to avoid refractory epilepsy [46]. Indeed, a recent randomized clinical trial demonstrated the significant efficacy of IVIGs in anti-LGI-1-IgG AIE compared to placebo, especially when corticosteroids are contraindicated [89]. Nevertheless, about 27% of patients might develop clinical relapses [83]. In these cases, second-line immunotherapy, such as cyclophosphamide or rituximab may be helpful [83].

### 3.6 Anti-Caspr2-IgG Autoimmune Encephalitis

Epilepsy and neuropsychiatric symptoms may characterize the clinical picture of Caspr2-IgG disease. Caspr2-IgG disease is a very heterogeneous immune-mediated disorder [90, 91]. It might present as LE in 42% of cases or as Morvan syndrome in 29% of cases. Anti-Caspr2-IgG LE may be pure or characterized by additional neurological features, as cerebellar impairment or pain in patients [91]. Morvan syndrome is characterized by an association of seizures, cognitive dysfunction, peripheral nerve hyperexcitability, disturbances of the autonomic nervous system and sleep [90, 91]. Patients of the male gender, aged around 60 years, are more likely to develop Caspr2-IgG disease [91]. The pathophysiologic mechanism underlying Caspr2-IgG disease is mediated by auto-Abs directed against Caspr2 proteins, which are transmembrane cell adhesion molecules, expressed in both central and peripheral nervous systems, especially in the initial part of the axons and juxtaparanodal regions of the Ranvier node [90]. Caspr2 proteins play a key role in regulating action potential and neuronal excitability, forming a complex with transient axonal glycoprotein-1 (TAG-1) and shaker-type VGKC (Kv1.1 and Kv1.2) [90]. Anti-Caspr2-IgG auto-Abs functionally block Caspr2 proteins, reorganizing their distribution at membranes without producing their internalization [90]. Their functional block creates a condition of hyperexcitability and network disturbances [90]. The ubiquitous localization of Caspr2 proteins makes the clinical picture extremely heterogeneous with a wide spectrum of clinical manifestations such as epilepsy, CI, pain, fever, myokymias, neuromyotonia, sleep abnormalities, movement disorders, psychiatric disturbances, dysautonomia [91] and episodic ataxia disturbances [92]. Epilepsy involves about 38% of patients, mainly manifesting as focal nonmotor seizures with impaired awareness in 70% of them, whereas the remaining percentage may display focal motor. Around half of them also present generalized tonic-clonic seizures [91]. As in other AIE, seizures may present in the early and late stages of the disease and could be associated with CI [91]. Moreover, transient epileptic amnesia has also been reported in Caspr2**-**IgG antibodies disturbances [93]. Patients with epileptic seizures or CI may have a normal brain MRI in 70% of cases or exhibit bilateral T2 hyperintensities in medial temporal lobes in 24% [91, 93]. ^18^F-FDG PET may depict hypermetabolism in the medial temporal lobe and diffuse cortical hypometabolism [94]. According to a previous study, anti-Caspr2-IgG could contribute to releasing glutamate from CA3-CA1 synapsis, enhancing the neuronal excitability of the hippocampal CA1 region and producing epileptiform activity [95]. Interestingly, patients with anti-Caspr2-IgG associated with mesial temporal lobe epilepsy (MTLE) and HS present infiltrating immune cells, confirming the role of inflammation in this disorder [95]. Another intriguing hypothesis correlates anti-Caspr2-IgG to the presence of chronic seizures and neuronal loss [95]. Pain syndrome might also be a symptom developed during the disease course, probably correlated to the involvement of small fibres [91]. During disease progression, patients may also present weight loss [91]. Around 19% of cases are associated with tumors, exhibiting an overlapping course of disease compared to patients without a neoplasm [91]. Most of the patients depict unremarkable CSF or may show mild pleocytosis [91]. Anti-Caspr2-IgG might occur in combination with anti-LGI-1-IgG auto-Abs, configuring a double-antibody positive autoimmune disease [96]. In such a case, the clinical spectrum may be wider [96]. Moreover, these patients are more likely to develop tumors, particularly thymoma [91]. Patients may benefit from first-line immunotherapy, including IVIGs, oral steroids, plasma exchange or a combination of these treatments, and from second-line immunotherapy, as cyclophosphamide or rituximab [91]. Tocilizumab, a monoclonal antibody inhibiting interleukin-6 (IL-6) receptor, is also useful in Caspr2-IgG disease [97]. Epileptic seizures may also particularly benefit from IVIGs [91].

### 3.7 Anti Gaba-B Receptor IGG Autoimmune Encephalitis

GABABR-IgG AIE, first described by Lancaster *et al.* [98], is an autoimmune disorder mediated by auto-Abs directed against the GABA-B receptor [99]. Anti-GABABR-IgG encephalitis represents the second most common AIE after NDMAR-AIE and is far more frequent than anti-GABA type A Receptor-IgG (GABAAR-IgG)AIE [99]. Recently, in a series of 242 patients with AIE, Yang and colleagues showed that 19% had anti-GABABR-IgG encephalitis [100]. The critical role of the GABAB receptor in regulating neurodevelopmental and cognitive functions is demonstrated by several conditions, such as epilepsy, autoimmune anti-GABABR-IgG encephalitis, autism spectrum disorders, fragile X syndrome, Down's syndrome, and Alzheimer's disease [101]. Patients in the sixth decade (mean age: 50 years) are more likely to develop this form of AIE. The onset of the disease is typically very rapid and may be preceded by prodromal symptoms, such as fever and non-specific. respiratory symptoms [100]. Clinical presentation may comprehend epileptic seizures, cognitive dysfunction, and behavior abnormalities. Epilepsy is the main clinical feature of anti-GABABR-IgG AIE, affecting about 100% of patients. Seizures semeiology includes both focal and tonic-clonic generalized seizures. About 40% of patients could develop a refractory SE [102]. EEG may reveal focal or diffuse slowing or epileptiform discharges. Anti-GABABR-IgG AIE is frequently associated with small cell lung cancer in about 50% of patients and has also been related to thymoma and duodenal neuroendocrine tumors [91, 103]. In Anti-GABABR-IgG encephalitis, it was demonstrated that the co-occurrence of Potassium Channel Tetramerization Domain-16 antibodies points towards a paraneoplastic origin [102]. The long-term prognosis of this AIE strictly depends on etiology: autoimmune cases had a better outcome than paraneoplastic ones, especially in neuropsychological functions [104]. Patients with anti-GABABR-IgG encephalitis could present persistent cognitive neuropsychiatric symptoms, as shown in a prospective study with a follow-up of 24 months [105]. Aggressive immunotherapy and tumor treatment could improve neurological outcomes [106]. A recent case series confirm the poor long-term prognosis of paraneoplastic anti-GABABR-IgG AIE [107, 108].

### 3.8 Anti-Gaba-A-Receptor-Igg Autoimmune Encephalitis

GABAAR-IgG AIE presents as a rapidly progressive encephalopathy associated with new-onset psychiatric symptoms, seizures or status epilepticus related to cortical and subcortical T2/FLAIR hyperintense lesions at brain MRI [108]. In such a disorder, auto-Abs are directed against the GABAAR, characterized by a pentameric structure with a central pore and five surrounding subunits expressing different functions. It modulates the neurotransmitter GABA and is targeted by several drugs, such as ASMs, sedatives and anxiolytic drugs. As anti-NMDAR-IgG AIE, auto-Abs-IgG against GABAAR induces their internalization and reduce their expression on the synaptic surface, producing a neuronal hyperxcitability [109]. The main features of anti-GABAAR-IgG encephalitis are seizures, neuropsychiatric deficits, and movement disorders. Seizures include both focal and generalized seizures. Although seizures are often refractory to ASMs, they response to immune treatment [109]. Patients also depict cognitive impairment, decreased level of consciousness, behavioral changes related to encephalopathy associated movement disorders. Paraneoplastic etiology occurs in about 20% of GABAAR-IgG forms [108]. Multifocal cortical-subcortical MRI abnormalities provide an important imaging clue to the diagnosis of anti-GABA-A encephalitis [109]. Immunotherapy may include first-line treatment with corticosteroids, plasma exchange, IVIGs, second-line treatment, such as rituximab, AZA, cyclosporine, and cyclophosphamide [109].

### 3.9 Anti-AMPA Receptor-igg Autoimmune Encephalitis

Anti-alpha-amino-3-hydroxy-5-methyl-4-isoxazole propionic acid receptor-IgG (AMPAR-IgG) encephalitis represents an immune-mediated disorder presenting as LE in most cases [110]. AMPAR is ubiquitous in CNS, particularly expressed in the hippocampus. They are cell-surface ionotropic receptors with a heterotetrameric structure which consists of a varying combination of 4 subunits, from GluA1 to GluA4. AMPAR exerts a crucial role in modulating synaptic transmission, memory, and learning [110]. The anti-AMPAR-IgG target specific domains of the correspondent receptor, decreasing the AMPAR number and, consequently, their functional activity, as demonstrated by immunocytochemistry findings [110]. AMPAR-IgG AIE predominantly affects female patients with a mean age of 60 years and is paraneoplastic in 64% of cases, related to thymoma, ovarian teratoma, and lung and breast cancers [111]. The main clinical features encompass LE, but patients may also display pure psychiatric syndromes, hyponatremia, diffuse encephalitis, dysautonomia, unilateral weakness and spasticity. A psychiatric onset is common in young patients [110]. Moreover, anti-AMPAR-IgG coexist with onconeural antibodies, such as SOX-1 antibodies, glutamic acid decarboxylase (GAD) antibodies, anti-neuronal auto-Abs, such as anti-NMDAR-IgG, and Hashimoto’s encephalopathy [109]. Furthermore, in AMPAR-IgG AIE, amnestic syndromes, in the absence of epileptic seizures and cognitive features, have also been reported, both as idiopathic and paraneoplastic forms [112]. CSF examination could demonstrate elevated levels of proteins, isolated or combined with lymphocytic pleocytosis [109]. EEG may be normal or detect focal and generalized epileptiform activity or slowing, as well as lateralized periodic discharges. Brain MRI showed unilaterally or bilaterally T2/FLAIR hyperintensities in the medial temporal lobe, basal ganglia, corpus callosum, frontobasal lobes, and parietal and frontal cortex [109]. Patients may develop rapid brain atrophy with a correspondent hypometabolism on ^18^F-FDG PET. Thus, ^18^F-FDG PET/MRI also provides useful information depicting both hypometabolic and hypermetabolic areas [111]. At the same time, ^18^F-FDG PET allows monitoring of the disease activity [113]. Different from anti-NMDAR-IgG AIE, in AMPA-IgG AIE, ^18^F-FDG-PET may detect an opposite frontal–occipital gradient of hypometabolism [114]. Unlike the anti-LG-1-IgG AIE, the histopathological finding demonstrates a neuronal loss in CA1 and CA4 subfields of the hippocampus affected by sclerosis. Other findings include activated microglia, astrogliosis, and inflammatory changes in meninges and brain parenchyma. Proteins suggestive of neurodegeneration were not revealed as well as complement activation [112]. Anti-AMPAR-IgG AIE could benefit from steroids, IVIGs, or plasma exchange treatments. Second-line immunotherapies include cyclophosphamide, rituximab, mycophenolate mofetil and AZA.

### 3.10 Anti-DPPX-IgG Autoimmune Encephalitis

The dipeptidyl-peptidase-like protein 6 (DPPX) is a subunit of Kv4.2 potassium channels expressed on gut and brain neurons. It increases the surface expression and channel conductance of Kv4.2 channels [115]. Anti-DPPX-IgG AIE was described in 2014 for the first time in patients with psychiatric symptoms, cognitive dysfunction, seizures, myoclonus, tremor dysautonomia, sleep disorders, brainstem symptoms, ocular disturbances [115] as opsoclonus [116] and cerebellar ataxia [117]. In addition, subsequent cases report anti-DPPX-IgG antibodies in patients with progressive encephalomyelitis with rigidity and myoclonus, progressive cognitive impairment with gait ataxia and rigidity preceded by gastrointestinal symptoms, [118] and in a patient with oro-bucco-lingual dyskinesias [119]. Other manifestations might include CNS hyperexcitability, developing myoclonus, hyperekplexia, tremor or seizures [115]. Despite the growing cases, the role of DPPX-IgG is still unclear. In some cases, DPPX may associate with lymphoma [120]. Anti-DPPX-IgG is probably directed against both CNS and enteric nervous system, leading to hyperexcitability of myenteric plessus and hippocampal neurons due to their decreased expression of DPPX proteins and Kv4.2 [121]. Thus, anti-DPPX-IgG encephalitis should be suspected in patients with AIE preceded by gastrointestinal symptoms, such as diarrhea, unexplained by a previous disease [97]. At the onset, the main ages are from 14 to 56 years [100]. The EEG may be unremarkable or shows diffuse slowing without epileptiform discharges [100]. Brain MRI might be normal or depict hyperintense hippocampus bilaterally [95, 100]. Interestingly, Johannes et colleagues reported a reduction of metabolism in caudate nuclei bilaterally and in the frontal cortex at ^18^F-FDG-PET, whereas other authors detect decreased metabolic activity in the bilateral mesial temporal lobes, which indicates that the limbic system is involved in anti-DPPX-IgG encephalitis [122]. However, in the literature, there are scarce findings on the ^18^F-FDG-PET usefulness in anti-DPPX-IgG encephalitis [123]. Patients could benefit from first-line and second-line immunotherapy [95].

### 3.11 Anti-Iglon5-IgG Autoimmune Encephalitis

The anti-immunoglobulin-like cell adhesion molecule (anti-IgLON5-IgG) disease represents an atypical form of AIE, manifesting mainly as a neurodegenerative disorder with sleep disturbances, bulbar dysfunction, progressive supranuclear palsy (PSP), CI and peripheral nervous system alterations [124]. IgLON5 is a neuronal adhesion molecule whose function is not fully understood. It is part of the superfamily of immunoglobulins, probably involved in neurite outgrowth. Although the mechanism underlying the anti-IgLON5-IgG disease is still unclear, a combined inflammatory and neurodegenerative process has been hypothesized, probably related to the binding of these antibodies to IgLON5 epitopes with a subsequent tau accumulation and neurodegeneration. Most histopathological studies detect the accumulation of tau isoforms in the absence of inflammatory infiltrates. However, according to a recent case report, the inflammatory process seems to play a critical role, especially in the early stage of the disease [125]. The anti-IgLON5-IgG disease predominantly affects patients with a median age of 60-66.5 years, although the onset may largely range between 33 and 83 years. About 59% of patients present supranuclear gaze palsy. About 77% of patients develop bulbar symptoms, gait disturbances, as ataxia, stiffness, dystonia, sleep dysfunctions, progressive CI and movement disorders, often mimicking PSP or multisystem atrophy (MSA) [126]. However, unlike PSP, anti-IgLON5-IgG disease is characterized by the predominant involvement of mesencephalic rather than pontine structures [127]. Compared to MSA, in anti-IgLON5-IgG disease, dysautonomia and orthostatic hypotension tend to be milder, whereas cerebellar or parkinsonian features are not predominant [128]. The association with tumors is unclear [126]. Patients affected by anti-IgLON5-IgG disease show pleocytosis on CSF analysis in 30% of cases and increased proteins in 50% [126]. Neurophysiological assessment may comprehend video-polysomnography to detect obstructive sleep apnea, and alterations of non-REM sleep, including parasomnias, vocalizations, limb movements, and REM sleep behavior disorder. EEG is normal in most cases. EMG might also evidence sensorimotor polyneuropathy [129]. MRI could be completely normal or show unspecific changes in 81-95% of cases [126]. Exceptionally brainstem, hippocampus and cerebellar atrophy have been reported [129]. Patients might scarcely benefit from immunotherapy with IV steroids or IVIGs, high dose corticosteroids, plasmapheresis, and second-line immunotherapy, such as azathioprine, rituximab or cyclophosphamide [129]. The response to treatment tends to be more effective in the early phase of the disease [129].

### 3.12 Encephalitis Associated with Intracellular Antibodies

Autoimmune LE presenting with neuropsychiatric features and seizures could be caused by onconeural antibodies targeting intracellular antigens [130]. In these cases, typically, oncologic diagnosis follows neurologic one and anti-tumor treatment seems more effective than immunotherapy [131]. Several intra-cellular antibodies were identified with varying tumor associations so that the oncologic work-up is often guided by neuronal antibodies [131]. Unlike surface auto-Abs, antibodies directed toward intracellular antigens do not appear to be pathogenetic: this could explain the typical lack of response by antibodies-depleting therapies [132]. The pathogenetic mechanism of this kind of encephalitis appears to be more related to cellular immunity, cytotoxic T-cell infiltration, and granzyme neuronal damage [133]. The anti-Hu antibodies are frequently associated with paraneoplastic encephalomyelitis and peripheral neuropathy [131]. Seizures are a usual clinical manifestation. Epilepsia partialis continua with facial twitching and arm jerking has been the most commonly described semiology [134]. Lung cancer can be found in 77-78% of patients with positive anti-Hu antibodies [131]. These auto-Abs recognize a family of RNA-binding proteins expressed in the nuclei of neurons and small cell lung cancer cells. The immune response against Hu-expressing tumor cells also destroys Hu-expressing neurons [135]. Ma1 and Ma2 have been identified as targets of autoimmune encephalitis. Ma1 is expressed in both the brain and testis, whereas Ma2 is predominantly expressed in the brain [136, 137]. The anti-Ma2/Ta antibodies react against neuronal nuclear proteins involved in RNA transcription and regulation of apoptosis. Ma2 antibody is most frequently associated with testicular cancer in the under-50 male population (53-77%) [136, 137]. Otherwise, it is also related to non-SCLC [138]. Dual Ma1/Ma2 is more common in women with breast cancer [139]. These antibodies are commonly associated with limbic and brainstem encephalitis [136]. In 30% of patients, seizures respond to tumor removal and immunotherapy [139]. Copresence of anti-Ma1 antibody and hypothalamic involvement is associated with a poor prognosis [137]. Recently, a novel autoantibody against anti-glial fibrillar acidic protein (GFAP) IgG has been identified. It is associated to relapsing and corticosteroid-responsive meningo-encephalitis [140]. Patients with a mean age of 40-50 years and female gender are more likely to develop it. The clinical spectrum is very heterogeneous, includes an acute, subacute or chronic onset of headache, rigor nucalis, movement disorders, ASMs-resistant epilepsy, cerebellar ataxia and optic neuritis. Nevertheless, meningoencephalitis with or without myelitis represents the most common clinical presentation [141]. GFAP AIE may also coexist with other immune systemic disorders. About one-third of cases are related to an underlying tumor. Far from the other paraneoplastic syndromes, GFAP autoimmune disease depicts a surprising response to immunotherapy, especially to corticosteroids [140, 141].

## 4 SYSTEMIC AUTOIMMUNE DISEASES AND EPILEPSY

**Fig. (1):**
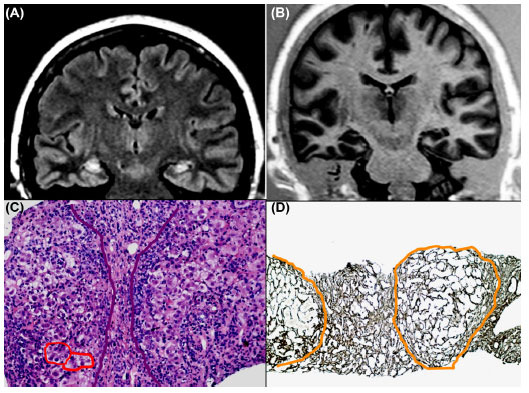
A 58-year-old woman with drug-resistant TLE and memory deficits. Brain MRI demonstrates bilateral hippocampal hyperintensities on T2-weighted images (**A**) with prominent left hippocampal atrophy (**B**). She also has an undifferentiated connective tissue disease and chronic auto-immune hepatitis, stage III. On liver biopsy, note the severe necro-inflammatory activity (red circles), fibrous septa and hepatocellular rosettes (Emat-Eos.20X) (**C**) and subversion of the acinar structure with segregation of noduliform areas of parenchyma (Gomori 10X) (**D**). Steroid therapy, in addition to ASMs, ameliorated control of epileptic seizures and resolved mild cognitive deficits. Inflammatory and immunological factors might contribute to epileptogenesis in TLE.

Patients with epilepsy have a higher prevalence of systemic autoimmune diseases than the general population, suggesting common mechanisms (Fig. **1**) [5, 142]. Patients with systemic autoimmune diseases also share high neuropsychiatric comorbidities. A cohort study shows a significant prevalence of epilepsy among patients with systemic autoimmune diseases [odds ratio 3.8; 95% confidence interval 3.6-4.0], especially in children [143]. The risk of seizures and neuropsychiatric features is elevated among all systemic autoimmune diseases but highest in systemic lupus erythematosus (SLE) and type 1 diabetes mellitus. Several mechanisms predisposing neuropsychiatric symptoms and epilepsy in patients with systemic autoimmune diseases are discussed, including genetic background, vascular disease, prothrombotic state, antineural antibodies, pro-inflammatory cytokines, and damage immune complexes-related [5-7, 144, 145]. A better comprehension of the role of these multifactorial predisposing factors may aid in the development of treatment strategies. Seizures and neuropsychiatric disorders are quite common in SLE and are also milestones for its diagnosis and classification. According to the 2019 European League Against Rheumatism/American College of Rheumatology Classification Criteria (EULAR/ACR) classification criteria for SLE, the “*neuropsychiatric domain*”, among the various clinical domains included for diagnosis, has an overall weight of 10 points, with a score for only seizure detection of 5 points [146]. Seizure crude prevalence in SLE patients ranges from 2% to 8% [147].

The incidence of seizures is most common in young females with SLE and the vast majority of patients present seizures within the first year from the onset of the disease [148]. SLE has been associated both with provoked and unprovoked seizures [149]. Many clinical semiologies of seizures related to SLE have been reported, including generalized tonic-clonic seizures, followed by focal seizures with or without impaired awareness [150, 151]. Thus, regarding the seizure type as clinical criteria for diagnosis of SLE, the EURAP/ACR criteria considered both primary generalized seizures and focal seizures [146]. In a cohort of 17 patients with coexisting SLE and epilepsy, Toyota and colleagues reported that 7/17 patients manifest an MTLE syndrome, suggesting that MTLE could also be a common presentation of SLE-related epilepsy [152]. About one-third of patients with definite SLE present “*neuropsychiatric lupus*” (NPSLE), a condition in which neurological and psychiatric features co-occur with SLE, without other clear precipitants [153]. The pathogenesis of seizures and neuropsychiatric features related to NPSLE is probably multifactorial. SLE, in fact, may affect the CNS by direct neuronal damage, injury to brain vessels or immune pathogenetic mechanisms, including the deposition of immune complexes [154]. Many risk factors associated with the development of seizures in SLE or NPSLE have been reported, including inflammatory disease activity. Thus, a high SLE Disease Activity Index score and diffuse multi-organ involvement have been associated with a shorter onset time and higher seizures recurrence [155]. The development of NPSLE may also be related to the penetrance of autoantibodies in the CNS [154], the disrupted BBB, or, as recently proposed, *via* the glymphatic pathway and intradural lymphatic network [156]. Several autoantibodies have been linked to the NPSLE pathogenesis, which might explain the intriguing combination of seizures and neuropsychiatric symptoms of this sub-set of patients. Antiphospholipid (aPL) antibodies, which were themselves linked to epilepsy, had a primary role. Patients with SLE and aPL antibodies are at twice the risk of developing NPSLE compared to aPLantibody negative people [157-159]. Among various aPL antibodies, patients with anti-β2 glycoprotein antibodies (anti-β2 GPI) IgG were 11 times more likely to exhibit seizures and nine times more likely to have tonic-clonic seizures compared to seronegative patients [160]. Furthermore, other auto-antibodies are found to be associated with seizures or NPSLE in SLE patients, including the anti-double-stranded DNA antibodies with cross-reactivity to subunits of NMDARs [161]. Moreover, anti-ribosomal P protein antibodies, which target brain antigens, are also linked to epilepsy in SLE patients [162]. The possible explanation for the occurrence of seizures and neuropsychiatric symptoms mediated by auto-Abs include both ischemic and non-ischemic mechanisms. Seizures might result from ischemic stroke due to thromboembolism secondary to a pro-thrombotic state mediated by aPL antibodies. The non-ischemic mechanisms are related to increased neuronal excitability by auto-Abs, through inhibition of the GABA receptor and depolarizing brain synapses [163]. Recently, Hopia and colleagues reported that the rates of cerebrovascular disease and psychosis were two- and three-fold elevated in NPSLE patients with epilepsy *versus* those without epilepsy, respectively [164]. aPL antibodies were also more found in patients with SLE and epilepsy rather than with SLE alone, further reinforcing the link between epilepsy, immunity and neuropsychiatric features [164]. Although neuropsychiatric features are a core clinical characteristic of SLE, the significance of psychiatric symptoms in patients with only ANA positivity should be carefully evaluated. In a recent retrospective review of about 5000 patients, the prevalence of NPSLE in patients with psychosis and ANAs positivity was very low (1.5%) [165]. Serum levels of inflammatory cytokines or either immune circulating molecules have been linked to SLE related epilepsy. Liang and colleagues recently demonstrated that expression of free interleukin-18 is increased in NPSLE, especially when associated with seizures [166]. The expression of high-mobility group box protein 1 and the toll-like receptor 4 has been also linked to NPSLE [167]. Moreover, the background genetic status seems to play a pivotal role: a potential genetic locus of susceptibility in chromosome 15q22 has been reported to be associated with seizures related to SLE [168]. This locus includes genes that encode for alpha and beta subunits of the neuronal nicotinic acetylcholine receptor, traditionally linked to epilepsy [168]. In a genome-wide association study, three single nucleotide polymorphisms of gene TREX1 are found to be associated with the development of seizures in SLE patients [169]. Furthermore, brain structural changes in SLE patients have been linked to the development of seizures or NPSLE [170]. It has been clarified that seizures, behavioural and cognitive changes in SLE patients may be related to cortical and sub-cortical lesions following vasculitic or mixed ischemic and inflammatory brain injury [170]. The evaluation of antibody serum status, brain MRI, EEG, and also CSF analysis should always be part of the diagnostic workup for NPSLE [171]. Management of NPSLE manifestations can be very challenging: first, there is no consensus regarding the treatment of seizures. According to Fisher’s definition of epilepsy [172], the EULAR guidelines recommend that anti-epileptic therapy should be started in patients with a high recurrence risk for seizures, like > 2 unprovoked seizures occurring in a 24h interval [146]. Regarding antiepileptic choice, anecdotal cases reported the selective effectiveness of topiramate for managing seizures related to SLE associated with anti-ribosomal P antibodies [173]. EULAR also recommends immunosuppression therapy for seizures associated with SLE relapses, including glucocorticoids as first-line and other immunosuppressive agents like cyclophosphamide or hydroxychloroquine [174]. Since hydroxychloroquine has an anti-thrombotic effect, its use is the gold standard for treating seizures related to aPL antibodies or thrombosis [175]. Optimization of NPLSE therapy can be very arduous since high-level clinical evidence is still lacking. A randomized control trial has compared intravenous cyclophosphamide infusions with bimonthly intravenous methylprednisolone in treating patients with severe NPSLE, showing a better response in the cyclophosphamide group [176]. Tokunaga *et al.* showed a rapid improvement of the clinical and imaging picture in ten patients with refractory NPSLE treated with rituximab [177]. Mok and colleagues conversely showed a significant benefit of lupus psychosis using oral cyclophosphamide for six months, followed by AZA maintenance therapy [178]. The effects of corticosteroid treatment on neuropsychiatric symptoms in NPSLE may be limited, if not deleterious. Lu and colleagues report only minimal efficacy in murine models [179], while Zhu and colleagues show that prednisone might disrupt rather than relieve metabolic changes in the frontal cortex and hypothalamus in lupus mice models [180]. Several studies report an association between type I diabetes (T1DM) and epilepsy. Patients with T1DM have a higher risk of developing seizures up to three times greater than controls [181, 182]. Both acute symptomatic seizures linked to hypoglycemic or hyperglycemic events or, conversely, recurrent unprovoked seizures have been described. In patients with unprovoked and T1DM comorbidity, the onset of T1DM usually precedes that of epilepsy [183]. Although a specific epileptic syndrome in T1DM is not delineated, some studies have suggested an association with TLE [184]. Regarding the comorbidity link suggested for this intriguing association, four pathophysiological mechanisms were considered: genetic predisposition, either innate and adaptative immune mediators, neuronal injury caused by metabolic disturbances and vascular disease [5]. Although genome-wide-linkage analysis has recently suggested common genetic bases from both autoimmune diseases and epilepsy [185], shared genetic factors in T1DM and epilepsy are only restricted to neonatal onset forms of diabetes mellitus presenting with seizures [186]. Immune system dysfunction in the innate or adaptative arm could also explain the link between T1DM and epilepsy. Several innate immunity inflammatory mediators could reduce the neuronal excitability threshold. In T1DM animal models with seizures, there are increased serum levels of cytokines and a more profound BBB dysfunction [187]. Concerning the role of the adaptive immune system, GAD65 antibodies were considered a risk factor for the development of seizures or neuropsychiatric symptoms in T1DM patients [188]. Furthermore, GAD-related epilepsy was also considered a distinct clinical picture with autoimmune epilepsy or encephalitis, in which immunotherapy can be effective if administered early, preventing permanent brain damage [189]. An important caveat was to suspect a neuropsychiatric or epileptic GAD65-related phenotype only detected with high serum GAD-Abs levels [190]. The clinical picture of T1DM goes far away from classic hyperglycemic manifestations, including neuropsychologic and neuroanatomic disabilities [191]. Aye and colleagues report a negative relationship between glycated hemoglobin levels compared to Wechsler Intelligence Scale for Children and hippocampal volume in children with type I diabetes [191]. General antiepileptic management does not differ in patients with T1DM and epilepsy from patients without a diagnosis of diabetes, although an optimal glycemic and metabolic control could produce a better outcome of seizures and neuropsychiatric symptoms [183]. Coeliac disease (CD) is a systemic chronic immune-mediated disorder elicitated by gluten in genetically susceptible patients. Many neurological presentations have been associated with CD, including seizures, and neuropsychiatric and cognitive disorders [192]. However, a causal relationship between these features is still a debated matter. The most valuable epidemiological association between epilepsy and CD comes from a cohort study of about 30000 subjects with a biopsy-confirmed disease, finding a 1.42-fold risk of epilepsy after a CD diagnosis over a mean follow-up of 10 years [193]. Also, in a cohort of young individuals with CD, Canova and colleagues show an increased risk of epilepsy, suggesting to perform coeliac antibodies screening on all patients with epilepsy without a clear etiology [194]. Conversely, some studies have suggested that about 40% of people with epilepsy could also present CD without gastrointestinal symptoms, but these studies were only based on coeliac autoantibodies detection, including the controversial “gluten sensitivity”, which can overestimate the diagnosis of comorbid CD [195]. The pathophysiological mechanisms triggering seizures in CD may include either gut dysfunction or cross-reactivity of systemic antibodies (transglutaminase antibodies) to neural antigens. Alterations of gut microbiota are quite common in CD. Recently microbiota composition is altered in patients with epilepsy, suggesting its role as a possible biomarker [196]. Furthermore, it is demonstrated that anti-transglutaminase antibodies can directly cross-react with neural antigens in the cerebellum, causing cerebellar ataxia [197]. Another proof of the association between epilepsy and CD comes from the well-recognized and widely accepted “CEC” (celiac disease, epilepsy and cerebral calcifications) syndrome [198]. In this disorder, patients often experience focal seizures with visual semiology and computed tomography (CT) scans show calcifications over the parieto-occipital lobes [198]. Antibodies anti-transglutaminase-6 have been thought to be a specific marker for neurological manifestations in CD, even in CEC patients, supporting a pathogenetic role [199], although their pathophysiological link with CEC has been recently questioned [200]. Our group has also demonstrated an association between silent CD and childhood focal epilepsy with occipital paroxysms, also suggesting to perform routinely coeliac screening in these patients [201]. Psychiatric disorders have been reported as a complication of CD in many patients, and several studies showed symptom improvement after starting a gluten-free diet [202]. The most important neuropsychiatric symptoms presented by CD patients were depression, apathy, anxiety, irritability and also eating disorders [192]. Clinicians should always be aware that seizures and neuropsychiatric symptoms could be part of the spectrum of CD manifestations. Recently, it was shown that children with CD and epilepsy had a high percentage of drug-resistant seizures compared to a control group with epilepsy only [203]. The gluten-free diet strategy can ameliorate seizure control in patients with epilepsy and CD, often resulting in a decrease in ASMs number or dosage [204]. Immunosuppression therapy could only be used in patients where a strict gluten-free diet alone has not been beneficial [204].

## 5 STEROID RESPONSIVE ENCEPHALOPATHY ASSOCIATED WITH AUTOIMMUNE THYROIDITIS

Hashimoto’s thyroiditis (HT), also known as chronic lymphocytic thyroiditis, is an autoimmune disorder clinically characterized by signs and symptoms of hypothyroidism and elevated levels of antithyroid peroxidase (TPO) antibodies in the serum of patients affected. Patients with HT have an increased risk for seizures and may also present with an encephalopathy called “steroid responsive encephalopathy associated with autoimmune thyroiditis” (SREAT) [205]. In 42% of patients with SREAT, a euthyroidism state is highlightable at the time of diagnosis [206]. About 50% of patients with SREAT manifest epileptic seizures [205]. Patients affected by SREAT present with acute symptomatic seizures, neuropsychiatric symptoms with high titers of anti-TPO antibodies and a clear dramatic response to corticosteroid therapy [205]. The great clinical improvement after steroid therapy has been considered as a confirmation criterion for a correct diagnosis. Titres of anti-TPO antibodies may help predict the response of immune treatment. The clinical course of this syndrome could be monophasic or either chronic relapsing, which often requires a long-term immunosuppression strategy [205]. Pfeuffer and colleagues showed that persistent intrathecal CD4+ T-cell activation, demonstrated in CSF of patients with SREAT, could be a recent promising biomarker for distinguishing between these two different clinical courses, with clear high prognostic impact [207]. Current diagnostic criteria for SREAT include: encephalopathy with seizures, mild or subclinical thyroid disease, normal brain imaging or with unspecific alterations, presence of high anti-TPO Abs levels and a reasonable exclusion of alternative causes [35]. Pediatric patients with SREAT could not have evidence of thyroid disease, but only cognitive regression with deficits in one or more other neuropsychiatric domains in the setting of antithyroid antibodies [208]. Treatment consisted of steroids in the acute phase with rapid improvement in symptoms. Maintenance therapy, often unnecessary, consists of rituximab, IVIgs or AZA.

## 6 INFLAMMATION AND EPILEPTOGENESIS: A BIDIRECTIONAL RELATIONSHIP

**Fig. (2):**
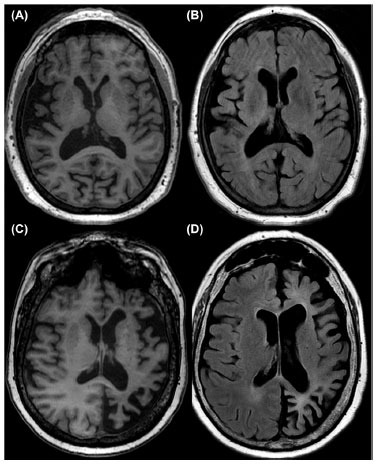
Brain MRI of two sisters with hemiconvulsion-hemiplegia syndrome (IHHS). Note the cortico-subcortical atrophy of the right hemisphere (**A**, **B**) in the 64-year-old proband, while the brain atrophy was contralateral and limited to the left hemisphere (**C**, **D**) in her 58-year-old sister.

The expanding insight into the complex spectrum of AIE and immune-mediated systemic disorders demonstrates the critical role of inflammation and immunity in developing epileptogenesis and related neuropsychiatric manifestations. However, it is not clear-cut whether inflammation acts as a cause or effect in epileptogenesis, leading to the hypothesis of a bidirectional relationship between these entities. In a broader perspective, the role of inflammation in epileptogenesis has also been investigated in epilepsy [209-212]. Animal models and histopathologic studies widely revealed elevated levels of pro-inflammatory molecules and cytokines, like interleukin-1β, tumor necrosis factor-α and IL-6, with activated microglia and astrocytes, in chronic drug-resistant epilepsy, in TLE and SE [209-212]. In immune-mediated conditions, immunity is the etiological factor of the epileptogenic process. Conversely, in epilepsy and SE, the inflammatory response may represent the consequence of a pro-epileptogenic event. In such conditions, the release of pro-inflammatory molecules further promotes the recurrence of epileptic activity [209, 210]. Several studies demonstrate that SE and epileptic seizures produce a pro-inflammatory response, further determining a recurrence of epileptic seizures. Inflammation contributes to different stages of epileptogenesis, persisting during both seizure recurrence and freedom [209, 210]. Epileptogenesis and inflammation are bidirectionally connected in some devastating clinical presentations, such as new onset refractory status epilepticus (NORSE), fever-induced refractory epileptic syndrome (FIRES) and idiopathic hemiconvulsion-hemiplegia syndrome (IHHS) [213]. These clinical entities represent a spectrum of conditions characterized by inflammation and epilepsy [214]. Indeed, they present as a refractory SE in previously healthy patients of all ages without a preexisting history of epilepsy, subsequently evolving into a chronic epileptic syndrome. In FIRES, a subgroup of NORSE, SE is preceded by fever [215]. This condition is characterized by a refractory SE with a previous febrile syndrome between 2 weeks and 24 hours, with or without fever at the onset of SE [215]. About 40% of NORSE recognize an autoimmune etiology. On the other hand, IHHS is a specific syndrome with fever and NORSE at the onset, presenting as unilateral motor seizures, associated with unilaterally abnormal imaging, evolving into hemiparesis lasting at least 24 hours, in the absence of infectious encephalitis [216]. Background genetic status might also influence clinical presentation of IHHS (Fig. **2**) [215-217].

In such conditions, a storm of proinflammatory and proconvulsant cytokines and chemokines has been demonstrated, remaining unclear whether inflammation is the cause or consequence of such conditions [216]. Animal, genetic, and pharmacological models demonstrated that pro-excitatory, pro-convulsive and inflammatory pathways are involved in epileptogenesis and subsequent neuronal damage [214]. The scarce response to ASMs and the improvement after immune therapies further suggest the critical role of inflammation [215]. In addition, pharmacological models demonstrated that inhibitors of inflammatory cytokines might reduce brain hyperexcitability and seizures threshold, providing new perspectives in the treatment of epilepsy [209, 210]. ASMs can influence both cellular and adaptative immunity, modifying the expression of many mediators, including cytokines [218]. This regulatory effect could be due to a modulation in the activity of transcription factors, like nuclear factor-kappaB (NF-κB) [218]. Several ASMs could exert an anti-inflammatory mechanism. Glial cells, having a key role in the stabilization of the brain network and BBB, may present morphological and functional changes during epileptogenesis. ASMs may influence such changes on glial cells directly [219]. Moreover, several ASMs, as levetiracetam, gabapentin, topiramate, valproic acid, carbamazepine, and phenytoin, depicted both pro- and anti-inflammatory effects [219]. Conversely, it is well known that many ASMs can produce mild to severe hypersensitivity reactions [220]. Many ASMs, like carbamazepine or levetiracetam, could trigger the development of drug-induced lupus erythematosus [221, 222]. *In vitro* studies demonstrated that many ASMs, like valproate, carbamazepine, levetiracetam, lamotrigine, and topiramate, modulate cytokine production, shifting toward an anti-inflammatory response [223]. The effect of recurrent seizures on inflammatory cascade might not be fully separated from that of the ongoing anti-epileptic treatment. Therefore, it is convincible that immune mechanisms can trigger seizures and epilepsy, and, conversely, recurrent epileptic seizures can cause inflammation.

## 7 DISCUSSION

Many studies in patients or animal models support the link between immunity, epilepsy and neuropsychiatric disorders [7, 8]. Several clinical diseases, which recognize an autoimmune pathogenetic mechanism, clinically manifest with seizures and neuropsychiatric symptoms. The RE was one of the first epileptic syndromes for which an inflammatory nature has been postulated [9, 12, 17]. AIEs, manifesting with subacute onset of seizures, neuropsychiatric symptoms, and cognitive impairment, actually had a high economic burden on medical health [35, 49, 50]. AIE can be further classified based on their etiology, pathogenetic auto-Abs association, and response to treatment. According to their etiology, AIE can be categorized as properly autoimmune and paraneoplastic, these last associated with neoplasia [35, 49, 50]. Based on their auto-Abs association, AIE can be also classified into two recognized categories: those with auto-Abs directed against surface antigens or those with auto-Abs directed against neuronal intracellular antigens. This review discussed the main types of AEI belonging to the first category, like anti-NMDAR-IgG, anti-LGI1-IgG, anti-CASPR2-IgG, anti-GABABR-IgG, anti-GABAAR-IgG, anti-AMPA-IgG, anti-DPPX-IgG, anti-IGLON5 AIE. In these syndromes, early recognition and immunotherapy typically allow for good long-term outcomes [35, 49, 50]. AIE caused by auto-Abs targeting intracellular antigens is associated with tumors [131, 134]. In these cases, typically, oncologic diagnosis follows neurologic one and anti-tumor therapy seems more effective than immunotherapy [131]. Unlike surface antigens, antibodies directed toward intracellular antigens do not appear to be pathogenetic: this could explain the lack of response by antibodies-depleting therapies [132]. There is a bidirectional epidemiologic link between epilepsy with neuropsychiatric symptoms and systemic autoimmune diseases: patients with epilepsy present a higher risk for developing systemic autoimmune disease and vice versa [5, 142-145]. Several mechanisms are claimed to explain this intriguing association, like genetic status, innate or adaptative arms of the immune system with CNS involvement [5, 154, 162, 188, 195, 197, 205]. This review highlights the comorbidity between seizures and neuropsychiatric features in SLE, emphasizing NPSLE, T1DM, CD and HT. NPSLE is a nosographic entity that specifically combines psychiatric and neurological symptoms like seizures with SLE [153]. Interestingly, as previously discussed, many auto-Abs, proven to be associated with NPSLE, also carry a higher risk for seizures or epilepsy, suggesting a shared mechanism, probably dealing with CNS involvement [153, 154]. The association between epilepsy, alterations of cognitive and neuropsychiatric domains has been already clarified in type I diabetes mellitus, especially when associated with anti-GAD65 auto-Abs [188, 189]. Moreover, the existence of GAD65 related epilepsy, manifesting with seizures or with complex encephalitis, further reinforces this association [188, 189]. It was also demonstrated that patients with CD present a high percentage of drug-resistant epilepsy, compared to those with epilepsy only [203]. Finally, patients with HT could experience seizures or encephalopathy, now called SREAT, in which intravenous methylprednisone administration allows full restoration of seizures and neuropsychiatric symptoms [35, 205].

## CONCLUSION

This review discusses the close relationship between immunity, seizures and additional neuropsychiatric symptoms in three main groups of diseases: first, RE, characterized by unilateral inflammation of brain cortex. Thus, we have reviewed epilepsy and neuropsychiatric comorbidities in AIE and systemic autoimmune diseases. In all conditions described in this review, targeted immunotherapy strategies might improve neuropsychiatric symptoms and seizures. Early immunotherapy has also been proven to prevent further risk for seizure recurrence. Further studies are warranted to better clarify the role of multifactorial predisposing factors, which may aid in developing new treatment strategies.

## LIST OF ABBREVIATIONS

AIE: Autoimmune-Mediated Encephalitis
AMPAR: Alpha-amino-3-hydroxy-5-methyl-4-isoxazolepropionic acid receptor
ANA: Antinuclear Antibodies
anti-β2 GPI: Anti-β2 Glycoprotein Antibodies
APE: Antibody Prevalence in Epilepsy of Unknown Etiology Score
aPL: Antiphospholipid
ASMs: Anti-Seizures Medications
Auto-Abs: Auto-Antibodies
AZA: Azathioprine
BBB: Blood-Brain Barrier
Caspr2: Contactin-Associated Protein-like 2
CD: Coeliac Disease
CEC: Celiac Disease, Epilepsy and Cerebral Calcifications
CI: Cognitive Impairment
CNS: Central Nervous System
CSF: Cerebrospinal Fluid
CT: Computed Tomography
DPPX: Dipeptidyl-Peptidase-Like Protein 6
EEG: Electroencephalogram
EULAR/ACR: European League Against Rheumatism/American College of Rheumatology
FBDS: Facio-brachial Dystonic Seizures
FIRES: Febrile-Illness Related Epilepsy Syndrome
18F-FDG PET: 18F-Fluorodeoxyglucose-Positron Emission Tomography
GABA-B: Gamma Aminobutyric Acid-B
GAD65: Glutamic Acid Decarboxylase 65
HS: Hippocampal Sclerosis
HT: Hashimoto’s Thyroiditis
ICU: Intensive Care Unit
Ig LON5: Immunoglobulin-like Cell Adhesion Molecule 5
IHHS: Idiopathic Hemiconvulsion-Hemiplegia Syndrome
Il-6: Interleukin-6
ILAE: International League Against Epilepsy
IVIGs: Intravenous Immunoglobulins
LE: Limbic Encephalitis
LGI-1: Leucine-Rich Glioma Inactivated-1
MRI: Magnetic Resonance Imaging
MSA: Multisystem Atrophy
MTLE: Mesial Temporal Lobe Epilepsy
NF-κB: Nuclear Factor-kappaB
NLR: Neutrophil-to-lymphocyte Ratio
NMDAR: N-methyl-D-aspartate Receptor
NORSE: New-Onset Refractory Status Epilepticus
NPSLE: Neuropsychiatric Lupus
PSP: Progressive Supranuclear Palsy
RE: Rasmussen Encephalitis
SCLC: Small Cell Lung Cancer
SE: Status Epilepticus
SLE: Systemic Lupus Erythematosus
SREAT: Steroid Responsive Encephalopathy Associated with Autoimmune Thyroiditis
T1DM: Type 1 Diabetes Mellitus
TAG-1: Transient Axonal Glycoprotein-1
TLE: Temporal Lobe Epilepsy
TPO: Antithyroid Peroxidase
